# Association between PNI and all-cause mortality in ischemic stroke patients: a large-scale retrospective cohort study

**DOI:** 10.3389/fnut.2026.1769193

**Published:** 2026-04-02

**Authors:** Junmou Li, Zhenwei Wang, Xuerong Sun, Lin Li, Zhuping Sun

**Affiliations:** 1 Department of Rehabilitation Medicine, Dandong Central Hospital, China Medical University, Dandong, Liaoning, China; 2 Department of Cardiology, The First Affiliated Hospital of Zhengzhou University, Zhengzhou, China

**Keywords:** all-cause mortality, ischemic stroke, prognostic nutritional index, prognostic biomarker, risk stratification

## Abstract

**Objectives:**

This study aims to explore the correlation between the prognostic nutritional index (PNI) and all-cause mortality in patients diagnosed with ischemic stroke (IS).

**Methods:**

A single-center retrospective cohort study was conducted at Dandong Central Hospital, enrolling 1,152 consecutive patients with IS who were discharged from January to December 2024. Multivariate Cox regression models, subgroup analysis, sensitivity analysis, receiver operating characteristic (ROC) curve, and Kaplan–Meier survival analysis were employed to investigate the association between the PNI and all-cause mortality.

**Results:**

During a median follow-up period of 14.23 months, a total of 96 (8.3%) patients experienced all-cause mortality. Multivariate Cox regression analysis showed that after adjusting for multiple confounding factors, each 1-unit increase in PNI was associated with an 8.5% reduction in all-cause mortality risk (hazard ratio [HR] = 0.915, 95% confidence interval [CI]: 0.883–0.947, *p* < 0.001), each 1-standard deviation increase was associated with a 40.1% reduction in all-cause mortality risk (HR = 0.599, *p* < 0.001). Compared with the lowest PNI quartile (Q1, PNI ≤ 44.16), the Q2 (PNI: 44.16–44.75) had a 54.1% lower risk of all-cause mortality (HR = 0.459, *p* = 0.004), the Q3 (PNI: 44.75–51.30) had a 56.4% lower risk of all-cause mortality (HR = 0.436, *p* = 0.007), the Q4 (PNI > 51.30) had a 72.6% lower risk of all-cause mortality (HR = 0.274, *p* < 0.001). Multiple subgroup and sensitivity analyses further confirmed the robustness of these associations. Stratified analyses based on various cutoff values of PNI uniformly demonstrated that patients with higher PNI levels had a notably reduced risk of all-cause mortality compared to those with lower PNI levels. ROC curve analysis indicated that PNI had favorable predictive value for all-cause mortality (overall population, AUC = 0.710; male, AUC = 0.720; female, AUC = 0.703; all *p* < 0.001). Kaplan–Meier survival curve analysis revealed significant differences in cumulative all-cause mortality risk among different PNI groups, with higher PNI levels correlating with lower cumulative mortality risk (Log-rank *p* < 0.001).

**Conclusion:**

The PNI establishes itself as an independent prognostic biomarker in IS patients, with higher levels correlating with a lower all-cause mortality risk.

## 1 Introduction

Ischemic stroke (IS) remains a critical global public health concern. Despite a declining trend in age-standardized incidence and mortality rates, the absolute number of IS cases continues to rise worldwide, driven by global population aging and the high prevalence of modifiable and non-modifiable risk factors (1). Characterized by high mortality, disability, and recurrence rates, IS poses a major challenge to clinical management, and accurate prognostic assessment and risk stratification are essential to optimize long-term patient outcomes.

The prognostic nutritional index (PNI), an objective nutritional assessment indicator based on serum albumin levels and lymphocyte counts, is widely used for clinical prognosis evaluation as either a continuous or categorical variable. In recent years, PNI has attracted increasing attention in the field of cerebrovascular diseases due to its unique value in disease risk stratification and outcome prediction (2). Systematic reviews and meta-analyses across multiple disease cohorts have confirmed that low PNI levels are significantly associated with poor clinical outcomes and increased all-cause mortality, with this association validated in populations including those undergoing joint replacement surgery and critically ill patients (2, 3). For stroke, impaired nutritional status can exacerbate neurological damage and hinder functional recovery, suggesting that PNI may have significant predictive value for IS prognosis. Moreover, PNI shows superior clinical utility in evaluating the nutritional reserve and immune function of stroke patients: compared with single traditional nutritional indicators such as serum albumin or lymphocyte count alone, PNI integrates two core parameters to more comprehensively reflect the body’s nutritional-immune interaction status, providing a reliable reference for assessing disease severity (2, 3). In addition, PNI can serve as a biomarker for early identification of stroke patients at high risk of malnutrition-related complications, as targeted nutritional intervention has been proven to improve relevant functional outcomes in this population (4). Collectively, these findings highlight the potential core role of PNI, a readily accessible and cost-effective hematological parameter, in the comprehensive management of stroke. Prior studies have confirmed that low PNI is significantly associated with an increased risk of in-hospital mortality in AIS patients (5, 6), as well as elevated 30-day and 90-day mortality and adverse functional outcomes in patients with AIS, those undergoing endovascular treatment, and critically ill stroke populations (7, 8). In addition, PNI has also been shown to predict post-stroke neurological impairment, long-term recurrence risk, cognitive impairment, and post-stroke complications in different IS subgroups (9–12), and its predictive performance is comparable to or even more clinically convenient than other complex nutritional and inflammatory prognostic indices (13–15). However, most of these studies focused on short-term outcomes within 3 months after stroke onset, while evidence regarding the association between PNI and medium- to long-term all-cause mortality in IS patients after discharge remains scarce. Furthermore, the dose–response relationship between continuous PNI levels and long-term mortality risk has not been clearly elucidated in large-scale IS cohorts, and the robustness of this association across different clinical subgroups still needs to be systematically verified, which constitutes the key research gap addressed in this study. To fill this critical research gap, this study aims to investigate the correlation between PNI and all-cause mortality in IS patients, with the goal of providing a new theoretical basis and practical reference for the clinical development of targeted nutritional intervention strategies and optimization of long-term prognosis in IS patients.

## 2 Methods

### 2.1 Study design and participants

This single-center retrospective cohort study was conducted at Dandong Central Hospital, enrolling patients discharged with a diagnosis of IS who received inpatient treatment in the general wards, and none of the enrolled patients were critically ill. A total of 1,152 consecutive participants were recruited from January 2024 to December 2024 in accordance with the following inclusion and exclusion criteria. Inclusion criteria: (1) Patients diagnosed with IS by computed tomography (CT) or magnetic resonance imaging (MRI); (2) Patients with complete baseline clinical data, demographic information, and laboratory test results. Exclusion criteria: (1) Patients with severe hematological disorders; (2) Patients with severe hepatic or renal insufficiency; (3) Patients with advanced malignant tumors; (4) Patients with severe autoimmune diseases; (5) Patients receiving hormonal or anti-inflammatory therapy; (6) Patients lacking baseline blood routine examination indicators or serum albumin; (7) Patients who died during hospitalization; (8) Patients lost to follow-up. The study protocol was approved by the Ethics Committee of Dandong Central Hospital (Approval No: DDSZXYY-2025-48) and adhered to the principles of the Declaration of Helsinki. As this was a retrospective study with anonymized data analysis, the requirement for informed consent from patients was reviewed and waived by the Ethics Committee of Dandong Central Hospital.

### 2.2 Data collection and variable definitions

Data on demographic characteristics, anthropometric measurements, comorbidities, previous medication history, and blood biomarkers were collected for all enrolled patients through the hospital’s electronic medical record system. Demographic data included age, sex, smoking status, and drinking status history. Smoking was defined as a history of regular smoking or current active smoking (regardless of subsequent cessation), while alcohol consumption was defined as a history of regular alcohol intake or ongoing consumption (regardless of abstinence attempts). Anthropometric measurements included height, weight, body mass index (BMI), systolic blood pressure (SBP), and diastolic blood pressure (DBP). BMI was calculated as weight (in kilograms) divided by the square of height (in meters).

Comorbidities included hypertension, diabetes, hyperlipidemia, previous cerebral infarction, and the duration of cerebral infarction. Hypertension was defined as a documented prior diagnosis of hypertension, SBP ≥ 140 mmHg or DBP ≥ 90 mmHg during the current hospitalization, or ongoing antihypertensive medication use (16). Diabetes was defined as a confirmed prior diagnosis of diabetes, fasting blood glucose (FBG) ≥ 7.0 mmol/L, glycated hemoglobin (HbA1c) ≥ 6.5%, current receipt of antidiabetic medication, or blood glucose ≥ 11.1 mmol/L 2 h after an oral glucose tolerance test (17). Hyperlipidemia was defined as a documented prior diagnosis of hyperlipidemia, triglycerides ≥ 2.3 mmol/L, total cholesterol (TC) ≥ 6.2 mmol/L, or low-density lipoprotein cholesterol (LDL-C) ≥ 4.1 mmol/L during the current hospitalization (18). IS is defined by the following criteria: (1) A documented history of prior IS or transient ischemic attack (TIA); (2) Acute-onset focal neurological deficits (e.g., limb weakness, sensory disturbance, aphasia, visual field defect) persisting for ≥ 24 h or leading to death; (3) Evidence of acute ischemic lesions in the corresponding vascular territory on cranial computed tomography (CT) or magnetic resonance imaging (MRI); (4) Confirmation of ≥ 50% atherosclerotic stenosis or occlusion in major intracranial arteries via transcranial Doppler (TCD), computed tomography angiography (CTA), magnetic resonance angiography (MRA), or digital subtraction angiography (DSA); (5) Exclusion of alternative etiologies including cardiogenic embolism, arterial dissection, infectious/non-infectious vasculitis, and reversible cerebral vasoconstriction syndrome; (6) Presence of atherosclerotic vascular risk factors (e.g., hypertension, diabetes, hyperlipidemia, smoking history) and demonstration of intracranial atherosclerotic plaque features on high-resolution vessel wall MRI (19). A history of cerebral infarction was defined as a documented prior diagnosis of the condition. The duration of cerebral infarction was recorded as the time interval from the initial onset of cerebral infarction symptoms to the current hospitalization. Previous medication history included information on the use of antihypertensive drugs, antidiabetic drugs, lipid-lowering drugs, antiplatelet drugs, and other relevant medications. Blood biomarker data included FBG, uric acid, estimated glomerular filtration rate (eGFR), triglycerides, TC, LDL-C, and high-density lipoprotein cholesterol (HDL-C). All biomarkers were measured through standardized procedures: trained nursing staff in our hospital collected antecubital venous blood samples from patients who had fasted for at least 8 h. The samples were promptly transported to the hospital’s central laboratory, where laboratory technicians performed the measurements in accordance with established operational procedures.

### 2.3 Definition and grouping of prognostic nutritional index

PNI is a well-recognized composite indicator for nutritional status assessment calculated based on serum albumin levels and lymphocyte counts, which can reflect the body’s nutritional status and immune function. Its calculation formula follows clinical standard specifications: PNI = serum albumin (g/L) + 5 × peripheral blood lymphocyte count (×10^9^/L) (20). Notably, all parameters used for PNI calculation in this study were laboratory values measured within 24 h after patient admission, and the final PNI value was computed during the patient’s hospitalization. In this study, participants were first divided into 4 groups according to the quartiles of PNI in the study population: Q1 group (PNI ≤ 44.16, *n* = 288), Q2 group (PNI 44.16–47.75, *n* = 291), Q3 group (PNI 47.75–51.30, *n* = 285), and Q4 group (PNI > 51.30, *n* = 288). Meanwhile, to verify the robustness of the results, we further grouped PNI using tertiles (T1 ≤ 45.40, T2: 45.40–50.05, T3 > 50.05), median (47.75), mean (47.68), and optimal cutoff value (44.00) to further analyze its association with all-cause mortality.

### 2.4 Follow-up and outcome measures

The start of follow-up was the date of patient discharge, and the end of follow-up was the time of all-cause death or September 2025, whichever occurred first. We adopted a pre-specified multimodal follow-up strategy, which was exclusively developed and implemented in line with the study design and core research objectives: first, we carefully reviewed the hospital’s electronic outpatient and emergency medical records, as well as inpatient medical files of patients with post-discharge re-visits to our hospital; second, follow-up information on patients’ post-discharge status was collected from the patients themselves or their authorized family members via telephone follow-up. It should be noted that the hospital also conducts routine follow-up for discharged patients, but such follow-up does not cover the monitoring of patients’ adverse prognosis or mortality events. The primary outcome measure of this study was all-cause mortality, defined as death resulting from any disease or event. Based on the occurrence of all-cause mortality during the follow-up period, the study cohort was divided into the all-cause mortality group (*n* = 96) and the non-all-cause mortality group (*n* = 1,056).

### 2.5 Statistical analysis

All statistical analyses were performed using SPSS 27.0 software (IBM Corporation, Armonk, New York, United States). Categorical variables were presented as frequencies (percentages), and intergroup comparisons were conducted using the Chi-square test or Fisher’s exact test. Continuous variables were first tested for normality using the Shapiro–Wilk test. Since all continuous variables did not conform to a normal distribution, they were expressed as median (interquartile range). Intergroup comparisons among multiple groups were performed using the Kruskal-Wallis H test, and comparisons between two groups were performed using the Mann–Whitney U test. Univariate Cox regression analysis was first used to assess the correlation between each variable and all-cause mortality. Variables with *p* < 0.05 were selected to construct three multivariate Cox regression models: Model 1 was adjusted only for age; Model 2 was adjusted for age, hyperlipidemia, previous cerebral infarction, lipid-lowering drugs, and antiplatelet drugs; Model 3 was further adjusted for SBP, DBP, FBG, uric acid, and eGFR on the basis of Model 2. The significant association between PNI and all-cause mortality was further evaluated through the above models. Subsequently, 10 subgroups were formed based on 5 variables: age, smoking status, drinking status history, diabetes, and previous cerebral infarction, and the stratified association between PNI and all-cause mortality was re-evaluated. In the sensitivity analysis, patients without hypertension and those without hyperlipidemia were excluded separately, and the correlation between PNI and all-cause mortality was re-verified in these subgroups. Receiver operating characteristic (ROC) curves were used to further assess the predictive value of PNI for all-cause mortality in the total population, male patients, and female patients. Finally, Kaplan–Meier survival curves were used to evaluate the differences in cumulative all-cause mortality risk among different PNI groups. All statistical tests were two-tailed, and *p* < 0.05 was considered statistically significant.

## 3 Results

### 3.1 Baseline characteristics grouped by PNI quartiles

The baseline characteristics grouped by PNI quartiles were presented in Table 1. The total sample size was 1,152, with a median age of 70.00 years (interquartile range: 64.00–77.00), including 643 males accounting for 55.8% of the total population. Compared with the Q1 group (lowest PNI), the Q4 group (highest PNI) had a younger age, lower prevalence of previous cerebral infarction, higher rate of antiplatelet drug use, higher BMI, DBP eGFR, triglyceride, TC, and LDL-C levels (*p* < 0.05). Notably, with the increase in PNI quartiles, the all-cause mortality rate showed a significant downward trend (18.1% in Q1, 6.9% in Q2, 5.3% in Q3, 3.1% in Q4 group, *p* < 0.001).

**Table 1:** Baseline characteristics grouped by quartiles of PNI.

Variables	Total population	Q1	Q2	Q3	Q4	*p* value
N	1,152	288	291	285	288	
Age, years	70.00 (64.00, 77.00)	76.00 (69.00, 82.00)	70.00 (64.00, 76.00)	69.00 (63.00, 75.00)	67.00 (60.00, 73.00)	<0.001
Sex, n (%)						0.883
Male	643 (55.8)	164 (56.9)	166 (57.0)	156 (54.7)	157 (54.5)	
Female	509 (44.2)	124 (43.1)	125 (43.0)	129 (45.3)	131 (45.5)	
Smoking, n (%)	433 (37.6)	107 (37.2)	108 (37.1)	102 (35.8)	116 (40.3)	0.721
Drinking, n (%)	341 (29.6)	94 (32.6)	87 (29.9)	82 (28.8)	78 (27.1)	0.521
Hypertension, n (%)	1,045 (90.7)	254 (88.2)	268 (92.1)	256 (89.8)	267 (92.1)	0.216
Diabetes, n (%)	521 (45.2)	125 (43.4)	122 (41.9)	136 (47.7)	138 (47.9)	0.358
Hyperlipidemia, n (%)	1,066 (92.5)	259 (89.9)	268 (92.1)	272 (95.4)	267 (92.7)	0.094
Previous cerebral infarction, n (%)	625 (54.3)	176 (61.1)	155 (53.3)	151 (53.0)	143 (49.7)	0.042
Course of cerebral infarction, day	1.00 (0.19, 3.38)	1.00 (0.17, 3.00)	1.00 (0.19, 3.00)	1.00 (0.34, 3.50)	1.00 (0.19, 4.38)	0.214
Antihypertensive drugs, n (%)	819 (71.1)	194 (67.4)	204 (70.1)	209 (73.3)	212 (73.6)	0.299
Antidiabetic drugs, n (%)	420 (36.5)	101 (35.1)	100 (34.4)	112 (39.3)	107 (37.2)	0.606
Lipid-lowering drugs, n (%)	1,062 (92.2)	259 (89.9)	267 (91.8)	271 (95.1)	265 (92.0)	0.141
Antiplatelet drugs, n (%)	1,038 (90.1)	244 (84.7)	263 (90.4)	263 (92.3)	268 (93.1)	0.003
BMI, kg/m^2^	24.58 (22.49, 27.06)	24.47 (21.48, 26.02)	24.77 (22.50, 26.67)	24.77 (22.85, 26.57)	25.39 (23.66, 27.69)	<0.001
SBP, mmHg	151.00 (139.00, 166.00)	150.00 (137.00, 168.50)	152.00 (141.50, 166.50)	150.00 (138.00, 163.00)	150.00 (139.00, 163.50)	0.443
DBP, mmHg	84.00 (78.00, 91.00)	83.00 (76.00, 90.50)	85.00 (80.00, 92.00)	83.00 (77.00, 90.00)	85.50 (80.00, 93.00)	<0.001
FBG, mmol/L	5.69 (5.00, 7.31)	5.52 (4.92, 7.28)	5.70 (5.00, 7.17)	5.86 (5.04, 7.67)	5.74 (5.01, 7.28)	0.357
Uric acid, μmol/L	325.00 (266.00, 389.75)	320.00 (260.25, 400.25)	314.00 (250.00, 385.00)	326.00 (271.00, 387.50)	335.44 (279.25, 389.75)	0.139
eGFR, mL/min/1.73m^2^	101.54 (80.64, 122.41)	95.52 (69.08, 117.19)	103.33 (83.90, 128.08)	103.60 (84.77, 123.65)	103.55 (85.78, 121.50)	<0.001
Triglycerides, mmo/L	1.35 (0.98, 1.89)	1.16 (0.84, 1.67)	1.28 (0.96, 1.72)	1.47 (1.07, 1.99)	1.53 (1.12, 2.26)	<0.001
Total cholesterol, mmo/L	4.45 (3.66, 5.21)	4.26 (3.57, 4.89)	4.41 (3.56, 5.08)	4.50 (3.79, 5.30)	4.61 (3.81, 5.45)	<0.001
LDL-C, mmol/L	2.74 (2.11, 3.29)	2.61 (1.98, 3.05)	2.72 (2.07, 3.27)	2.77 (2.20, 3.35)	2.84 (2.26, 3.49)	<0.001
HDL-C, mmol/L	1.13 (0.95, 1.32)	1.13 (0.94, 1.30)	1.13 (0.96, 1.30)	1.14 (0.96, 1.34)	1.13 (0.94, 1.36)	0.851
All-cause mortality, n (%)						<0.001
Yes	96 (8.3)	52 (18.1)	20 (6.9)	15 (5.3)	9 (3.1)	
No	1,056 (91.7)	236 (81.9)	271 (93.1)	270 (94.7)	279 (96.9)	

Q1: ≤ 44.16; Q2: 44.16–47.75; Q3: 47.75–51.30; Q4: > 51.30. PNI, Prognostic Nutritional Index; BMI, Body mass index; SBP, Systolic blood pressure; DBP, Diastolic blood pressure; FBG, Fasting blood glucose; eGFR, Estimated glomerular filtration rate; LDL-C, Low-density lipoprotein cholesterol; HDL-C, High-density lipoprotein cholesterol.

### 3.2 Multivariate cox regression analysis of PNI and all-cause mortality

The results of multivariate Cox regression analysis were presented in Table 2. In Model 1 (adjusted only for age) and Model 2 (adjusted for age, hyperlipidemia, previous cerebral infarction, lipid-lowering drugs, and antiplatelet drugs), higher levels of PNI were significantly associated with a lower risk of all-cause mortality (*p* < 0.05). In the fully adjusted Model 3 further adjusted for SBP, DBP, FBG, uric acid, and eGFR, the above association remained robust: each 1-unit increase in PNI was associated with an 8.5% decrease in all-cause mortality risk (HR = 0.915, 95% CI: 0.883–0.947, *p* < 0.001); the HR for standardized PNI was 0.599 (95% CI: 0.490–0.733, *p* < 0.001); compared with the Q1 group, the HR for the Q2 group was 0.459 (95% CI: 0.269–0.784, *p* = 0.004), for the Q3 group was 0.436 (95% CI: 0.239–0.795, *p* = 0.007), and for the Q4 group was 0.274 (95% CI: 0.130–0.580, *p* < 0.001), with a significant trend test (*p* < 0.001).

**Table 2:** Multivariate cox regression analysis of PNI and all-cause mortality.

Variables	Model 1		Model 2		Model 3	
	HR (95% CI)	*p* value	HR (95% CI)	*p* value	HR (95% CI)	*p* value
PNI (continuous variable)						
PNI	0.906 (0.873, 0.940)	<0.001	0.918 (0.884, 0.952)	<0.001	0.915 (0.883, 0.947)	<0.001
Standardized PNI	0.567 (0.459, 0.701)	<0.001	0.610 (0.492, 0.755)	<0.001	0.599 (0.490, 0.733)	<0.001
PNI (categorical variable)						
Q1	Ref			Ref		
Q2	0.504 (0.298, 0.853)	0.011	0.528 (0.310, 0.897)	0.018	0.459 (0.269, 0.784)	0.004
Q3	0.420 (0.233, 0.760)	0.004	0.457 (0.252, 0.832)	0.010	0.436 (0.239, 0.795)	0.007
Q4	0.290 (0.140, 0.605)	<0.001	0.309 (0.147, 0.651)	0.002	0.274 (0.130, 0.580)	<0.001
P for trend		<0.001		0.002		<0.001

Model 1: adjusted for age only; Model 2: adjusted for age, hyperlipidemia, previous cerebral infarction, lipid-lowering drugs, and antiplatelet drugs; Model 3: adjusted for age, hyperlipidemia, previous cerebral infarction, lipid-lowering drugs, antiplatelet drugs, SBP, DBP, FBG, uric acid, and eGFR. PNI, Prognostic nutritional index; SBP, Systolic blood pressure; DBP, Diastolic blood pressure; FBG, Fasting blood glucose; eGFR, Estimated glomerular filtration rate.

### 3.3 Subgroup analysis of PNI and all-cause mortality

As shown in Table 3, subgroup analysis results showed that the negative correlation between PNI and all-cause mortality risk remained robust across all stratified populations: Age stratification: < 70 years old group (each 1-unit increase in PNI, HR = 0.918, *p* = 0.021); ≥ 70 years old group (HR = 0.910, *p* < 0.001); among them, the HR for Q4 vs. Q1 in the ≥ 70 years old group was 0.290 (*p* = 0.009). Smoking stratification: Smoker group (HR = 0.856, *p* < 0.001); Non-smoker group (HR = 0.932, *p* = 0.002); among them, the HR for Q4 vs. Q1 in the smoker group was 0.084 (*p* = 0.017). Drinking status stratification: Drinker group (HR = 0.878, *p* = 0.003); Non-drinker group (HR = 0.923, *p* < 0.001); among them, the HR for Q2 vs. Q1 in the drinker group was 0.116 (*p* = 0.005). Diabetes stratification: Diabetic group (HR = 0.902, *p* < 0.001); Non-diabetic group (HR = 0.916, *p* = 0.001); among them, the HR for Q4 vs. Q1 in the diabetic group was 0.180 (*p* = 0.002). Previous cerebral infarction stratification: With previous cerebral infarction group (HR = 0.887, *p* < 0.001); without previous cerebral infarction group (HR = 0.937, *p* = 0.023); among them, the HR for Q3 vs. Q1 in the group with previous cerebral infarction was 0.374 (*p* = 0.010).

**Table 3:** Subgroup analysis of the correlation between PNI and all-cause mortality.

Subgroups	Q2 vs. Q1	Q3 vs. Q1	Q4 vs. Q1	PNI	Standardized PNI
	HR (95% CI) *p* value	HR (95% CI) *p* value	HR (95% CI) *p* value	HR (95% CI) *p* value	HR (95% CI) *p* value
Age					
< 70 years	0.451 (0.134, 1.518) 0.198	0.213 (0.049, 0.927) 0.039	0.173 (0.040, 0.748) 0.019	0.918 (0.853, 0.987) 0.021	0.610 (0.400, 0.929) 0.021
≥ 70 years	0.467 (0.253, 0.862) 0.015	0.528 (0.272, 1.024) 0.059	0.290 (0.116, 0.730) 0.009	0.910 (0.872, 0.951) < 0.001	0.583 (0.454, 0.749) < 0.001
Smoking					
Yes	0.175 (0.058, 0.522) 0.002	0.562 (0.224, 1.412) 0.220	0.084 (0.011, 0.642) 0.017	0.856 (0.789, 0.928) < 0.001	0.408 (0.256, 0.651) < 0.001
No	0.701 (0.372, 1.320) 0.272	0.383 (0.171, 0.860) 0.020	0.434 (0.134, 0.798) 0.049	0.932 (0.890, 0.975) 0.002	0.665 (0.512, 0.864) 0.002
Drinking					
Yes	0.116 (0.026, 0.518) 0.005	0.576 (0.207, 1.602) 0.291	0.277 (0.070, 1.098) 0.068	0.878 (0.806, 0.957) 0.003	0.474 (0.289, 0.777) 0.003
No	0.685 (0.377, 1.246) 0.215	0.368 (0.172, 0.788) 0.010	0.283 (0.113, 0.711) 0.007	0.923 (0.884, 0.965) < 0.001	0.633 (0.492, 0.813) < 0.001
Diabetes					
Yes	0.273 (0.117, 0.633) 0.003	0.374 (0.163, 0.858) 0.020	0.180 (0.060, 0.538) 0.002	0.902 (0.857, 0.949) < 0.001	0.552 (0.412, 0.738) < 0.001
No	0.592 (0.287, 1.220) 0.155	0.504 (0.205, 1.243) 0.137	0.325 (0.111, 0.950) 0.040	0.916 (0.869, 0.966) 0.001	0.605 (0.446, 0.820) 0.001
Previous cerebral infarction					
Yes	0.454 (0.232, 0.887) 0.021	0.374 (0.177, 0.792) 0.010	0.308 (0.116, 0.815) 0.018	0.887 (0.843, 0.934) < 0.001	0.503 (0.376, 0.674) < 0.001
No	0.547 (0.215, 1.391) 0.205	0.693 (0.231, 2.083) 0.514	0.212 (0.061, 0.738) 0.015	0.937 (0.886, 0.991) 0.023	0.688 (0.498, 0.949) 0.023

Subgroup analysis adjusted for age, hyperlipidemia, previous cerebral infarction, lipid-lowering drugs, antiplatelet drugs, SBP, DBP, FBG, uric acid, and eGFR. PNI, Prognostic nutritional index; SBP, Systolic blood pressure; DBP, Diastolic blood pressure; FBG, Fasting blood glucose; eGFR, Estimated glomerular filtration rate.

### 3.4 Multivariate cox regression analysis of PNI and all-cause mortality after excluding patients without hypertension or hyperlipidemia

As shown in Table 4, after excluding patients without hypertension, multivariate Cox regression analysis showed that in the fully adjusted Model 3, each 1-unit increase in PNI was associated with a 7.4% decrease in all-cause mortality risk (HR = 0.926, 95% CI: 0.899–0.953, *p* < 0.001); compared with the Q1 group, the all-cause mortality risk in the Q4 group decreased by 70.6% (HR = 0.294, 95% CI: 0.138–0.626, *p* < 0.001).

**Table 4:** Multivariate cox regression analysis of PNI and all-cause mortality: exclude patients without hypertension or hyperlipidemia.

Variables	Model 1		Model 2		Model 3	
	HR (95% CI)	*p* value	HR (95% CI)	*p* value	HR (95% CI)	*p* value
With hypertension						
PNI (continuous variable)						
PNI	0.913 (0.879, 0.948)	<0.001	0.936 (0.910, 0.963)	<0.001	0.926 (0.899, 0.953)	<0.001
Standardized PNI	0.591 (0.476, 0.734)	<0.001	0.684 (0.580, 0.806)	<0.001	0.643 (0.544, 0.760)	<0.001
PNI (categorical variable)						
Q1	Ref		Ref		Ref	
Q2	0.505 (0.293, 0.870)	0.014	0.519 (0.300, 0.896)	0.019	0.479 (0.276, 0.831)	0.009
Q3	0.412 (0.223, 0.760)	0.005	0.451 (0.243, 0.838)	0.012	0.441 (0.237, 0.823)	0.010
Q4	0.301 (0.144, 0.631)	0.001	0.321 (0.152, 0.679)	0.003	0.294 (0.138, 0.626)	<0.001
P for trend		0.001		0.003		0.001
With hyperlipidemia						
PNI (continuous variable)						
PNI	0.923 (0.895, 0.953)	<0.001	0.920 (0.892, 0.949)	<0.001	0.910 (0.882, 0.938)	<0.001
Standardized PNI	0.632 (0.527, 0.758)	<0.001	0.620 (0.519, 0.740)	<0.001	0.580 (0.485, 0.694)	<0.001
PNI (categorical variable)						
Q1	Ref		Ref		Ref	
Q2	0.564 (0.321, 0.992)	0.047	0.568 (0.322, 1.004)	0.052	0.508 (0.286, 0.900)	0.020
Q3	0.464 (0.249, 0.866)	0.016	0.472 (0.253, 0.884)	0.019	0.461 (0.245, 0.866)	0.016
Q4	0.240 (0.099, 0.580)	0.002	0.236 (0.097, 0.574)	0.001	0.210 (0.086, 0.516)	<0.001
P for trend		0.003		0.004		0.001

Model 1: adjusted for age only; Model 2: adjusted for age, hyperlipidemia, previous cerebral infarction, lipid-lowering drugs, and antiplatelet drugs; Model 3: adjusted for age, hyperlipidemia, previous cerebral infarction, lipid-lowering drugs, antiplatelet drugs, SBP, DBP, FBG, uric acid, and eGFR. PNI, Prognostic nutritional index; SBP, Systolic blood pressure; DBP, Diastolic blood pressure; FBG, Fasting blood glucose; eGFR, Estimated glomerular filtration rate.

After excluding patients without hyperlipidemia, multivariate Cox regression analysis showed that in the fully adjusted Model 3, each 1-unit increase in PNI was associated with a 9.0% decrease in all-cause mortality risk (HR = 0.910, 95% CI: 0.882–0.938, *p* < 0.001); compared with the Q1 group, the all-cause mortality risk in the Q4 group decreased by 79.0% (HR = 0.210, 95% CI: 0.086–0.516, *p* < 0.001).

### 3.5 Multivariate cox regression analysis of PNI and all-cause mortality based on different PNI cutoff values

As shown in Table 5, grouping analysis using different PNI cutoff values all showed that the all-cause mortality risk in the high PNI group was significantly lower than that in the low PNI group: Tertiles grouping: Compared with the T1 group (PNI ≤ 45.40), the HR for the T3 group (PNI > 50.05) in Model 3 was 0.357 (95% CI: 0.188–0.679, *p* = 0.002), with a significant trend test (*p* = 0.002). Median grouping: High PNI group (PNI > 47.75) vs. Low PNI group (PNI ≤ 47.75), HR = 0.501 (95% CI: 0.309–0.813, *p* = 0.005) in Model 3. Mean grouping: High PNI group (PNI > 47.68) vs. Low PNI group (PNI ≤ 47.68), HR = 0.511 (95% CI: 0.317–0.825, *p* = 0.006) in Model 3. Optimal cutoff value grouping: High PNI group (PNI > 44.00) vs. Low PNI group (PNI ≤ 44.00), HR = 0.380 (95% CI: 0.246–0.587, *p* < 0.001) in Model 3, with the strongest association observed at this cutoff value.

**Table 5:** Multivariate cox regression analysis of PNI and all-cause mortality: Grouped according to different PNI cutoff points.

Variables	Model 1		Model 2		Model 3	
	HR (95% CI)	*p* value	HR (95% CI)	*p* value	HR (95% CI)	*p* value
PNI (Tertiles)						
T1	Ref		Ref		Ref	
T2	0.585 (0.362, 0.946)	0.029	0.617 (0.380, 1.000)	0.050	0.542 (0.333, 0.883)	0.014
T3	0.374 (0.200, 0.700)	0.002	0.394 (0.209, 0.744)	0.004	0.357 (0.188, 0.679)	0.002
P for trend		0.003		0.008		0.002
PNI (Median)						
Low PNI (≤ 47.75)	Ref		Ref		Ref	
High PNI (> 47.75)	0.476 (0.295, 0.767)	0.002	0.507 (0.313, 0.820)	0.006	0.501 (0.309, 0.813)	0.005
PNI (Mean)						
Low PNI (≤ 47.68)	Ref		Ref		Ref	
High PNI (> 47.68)	0.481 (0.301, 0.771)	0.002	0.516 (0.320, 0.831)	0.006	0.511 (0.317, 0.825)	0.006
PNI (Optimal cutoff value)						
Low PNI (≤ 44.00)	Ref		Ref		Ref	
High PNI (> 44.00)	0.392 (0.256, 0.599)	<0.001	0.423 (0.274, 0.652)	<0.001	0.380 (0.246, 0.587)	<0.001

Model 1: adjusted for age only; Model 2: adjusted for age, hyperlipidemia, previous cerebral infarction, lipid-lowering drugs, and antiplatelet drugs; Model 3: adjusted for age, hyperlipidemia, previous cerebral infarction, lipid-lowering drugs, antiplatelet drugs, SBP, DBP, FBG, uric acid, and eGFR. T1: ≤ 45.40; T2: 45.40–50.05; T4: > 50.05. PNI, Prognostic nutritional index; SBP, Systolic blood pressure; DBP, Diastolic blood pressure; FBG, Fasting blood glucose; eGFR, Estimated glomerular filtration rate.

### 3.6 Visualization analysis of the association between PNI and all-cause mortality

**Figure 1:**
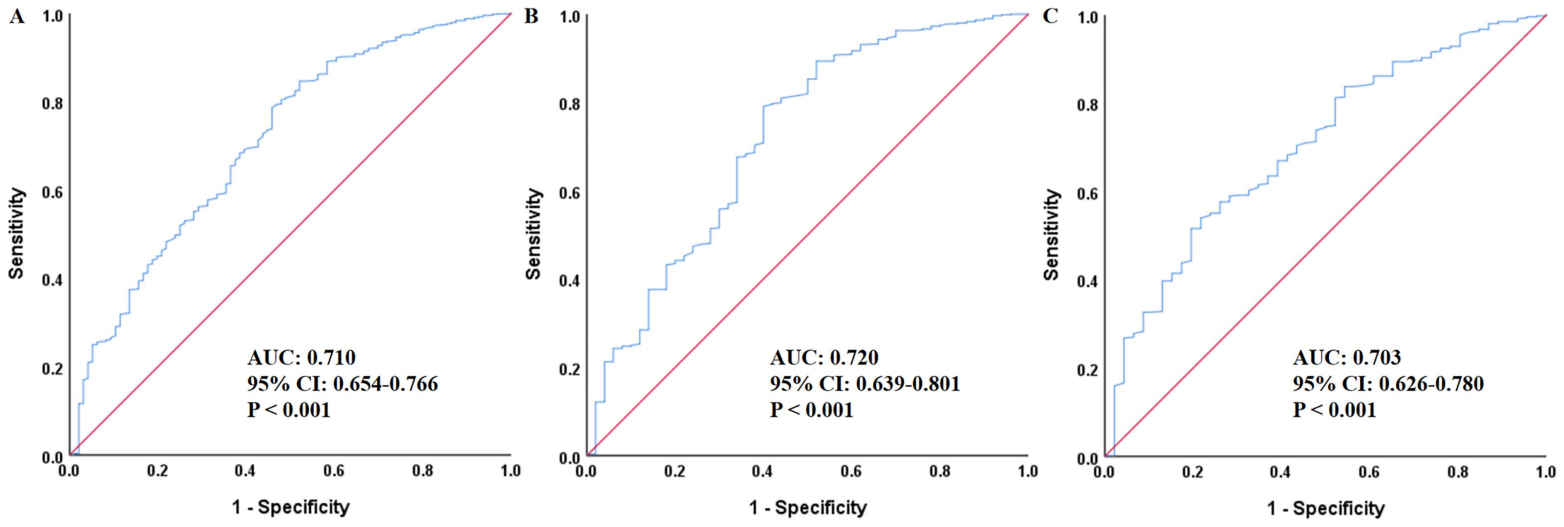
ROC curves assessing the predictive value of PNI for all-cause mortality in the total population **(A)**, men **(B)**, and women **(C)**. PNI, Prognostic nutritional index; ROC, receiver operating characteristic; AUC, area under the curve.

**Figure 2:**
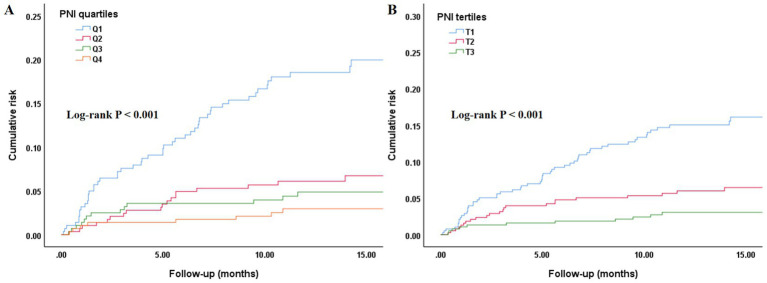
Kaplan–Meier curve assessing the differences in the cumulative risk of all-cause mortality among different SII groups. **(A)** PNI quartiles and all-cause mortality; **(B)** PNI tertiles and all-cause mortality. PNI, prognostic nutritional index.

**Figure 3:**
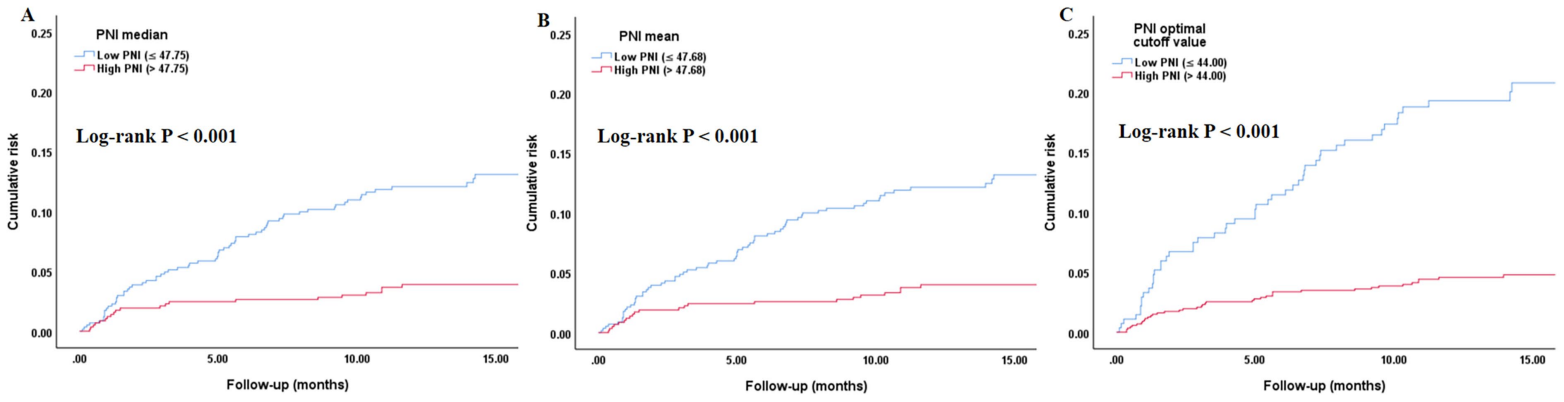
Kaplan–Meier curve assessing the differences in the cumulative risk of all-cause mortality among different SII groups. **(A)** PNI median (47.75) and all-cause mortality; **(B)** PNI mean (47.68) and all-cause mortality; **(C)** PNI optimal cutoff value (44.00) and all-cause mortality. PNI, prognostic nutritional index.

As shown in Figure 1, ROC curve analysis indicated that PNI had good predictive value for all-cause mortality risk, which was significant in the total population, male, and female subgroups: the area under the curve (AUC) for the total population was 0.710 (95% CI: 0.654–0.766, *p* < 0.001), for males was 0.720 (95% CI: 0.639–0.801, *p* < 0.001), and for females was 0.703 (95% CI: 0.626–0.780, *p* < 0.001). As shown in Figures 2, 3, regardless of grouping by the median, mean, or optimal cutoff value of PNI, there were significant differences in the cumulative risk of all-cause mortality among the different PNI groups (*p* < 0.001).

## 4 Discussion

The results found in this study demonstrated that the PNI was an independent protective factor for all-cause mortality after discharge, with a significant dose–response relationship between elevated PNI levels and reduced long-term mortality risk. This association remained robust after adjusting for multiple confounding factors, and was further validated by consistent results in subgroup analyses, sensitivity analyses with different exclusion criteria and grouping strategies, ROC curve evaluation, and Kaplan–Meier survival analysis. The mechanisms underlying the protective effect of higher PNI may involve furnishing essential nutritional support for neuronal recovery and vascular remodeling, as well as modulating post-stroke inflammatory responses via intact cellular immunity; the more prominent prognostic value observed in specific subgroups may be attributed to the fact that smokers with nicotine-induced endothelial dysfunction and patients with prior cerebral infarction with underlying vascular lesions exhibit weaker resilience to ischemia-related damage, thereby rendering the nutritional and immune support reflected by PNI more critical for survival (21, 22). Our findings complement and extend the current evidence on the prognostic role of PNI in IS. As outlined in the introduction, existing research has mostly focused on the short-term prognostic value of PNI within 3 months of stroke onset, while data on its association with medium- to long-term all-cause mortality in discharged IS patients remains scarce. Our study confirmed the stable and independent predictive performance of PNI for all-cause mortality during a median follow-up of 14.23 months, filling this key evidence gap. In addition, unlike most previous studies that analyzed PNI with a single predefined cutoff value (5, 7, 8), we systematically evaluated the linear dose–response relationship between PNI and mortality risk through continuous variable analysis and multiple grouping strategies, which can provide more graded and detailed reference for clinical risk stratification. Furthermore, our comprehensive subgroup analyses confirmed the robustness of this association across populations with different clinical characteristics, and further identified that the protective effect of higher PNI was more prominent in smokers and patients with previous cerebral infarction. This finding helps to identify high-risk IS patients who may benefit more from early nutritional assessment and targeted immunonutrition intervention in routine clinical practice. Existing evidence suggests that the following pathophysiological mechanisms may mediate the association between PNI and all-cause mortality in patients with IS. The core lies in PNI’s comprehensive reflection of nutritional reserve and immune function, which modulates the progression of local brain injury and systemic responses following IS, ultimately influencing patient prognosis. First, neuroinflammation and immune dysregulation represent the central pathological links mediating this association. Following IS onset, cerebral ischemia and hypoxia rapidly activate microglia and recruit peripheral immune cells to infiltrate the infarct site, triggering the release of pro-inflammatory cytokines such as tumor necrosis factor-*α* (TNF-α), interleukin-1β (IL-1β), and interleukin-6 (IL-6). These cytokines expand infarct volume by exacerbating neuronal edema and disrupting synaptic connections (23–25). Patients with low PNI exhibit impaired immune cell function (particularly lymphocytes) (26), which attenuates anti-inflammatory and reparative capacities, leading to uncontrolled inflammation, accelerated neuronal death, and increased risk of secondary infection—all contributing to elevated mortality (27). Second, oxidative stress injury plays a key regulatory role in the association between PNI and mortality in IS patients. The ischemia–reperfusion process post-IS induces excessive production of reactive oxygen species (ROS), which attack lipids, proteins, and nucleic acids, causing lipid peroxidation of cell membranes, protein denaturation, and DNA damage, directly promoting neuronal apoptosis (23–25). Patients with low PNI have reduced serum albumin levels, significantly impairing antioxidant and free radical-scavenging capacities, and thus failing to neutralize excess ROS generated post-IS. This leads to persistent exacerbation of oxidative stress injury, accelerating neuronal death and infarct expansion (24, 25). Third, apoptosis and excitotoxicity jointly contribute to pathogenesis. Energy failure induced by IS triggers excessive glutamate release, overactivating N-methyl-D-aspartate (NMDA) receptors and causing calcium overload, which activates apoptotic pathways. The malnutrition associated with low PNI reduces mitochondrial function and energy metabolism efficiency in neurons, lowering their tolerance to excitotoxicity (23–25). Additionally, low PNI may alter the balance of Bcl-2/Bax family proteins, further enhancing the activation of intrinsic and extrinsic apoptotic pathways induced by ischemia-hypoxia, thereby promoting programmed neuronal death (23–25). Fourth, blood–brain barrier (BBB) disruption and elevated risk of secondary complications exert synergistic damaging effects. Post-IS inflammatory factors (e.g., matrix metalloproteinases [MMPs]) and oxidative stress impair BBB tight junctions. In patients with low PNI, insufficient serum albumin reduces plasma colloid osmotic pressure, further exacerbating vasogenic cerebral edema. BBB disruption, in turn, promotes inflammatory cell infiltration and hemorrhagic transformation, forming a vicious cycle (24, 25). Moreover, systemic malnutrition and immunosuppression from low PNI significantly increase the risk of complications such as pressure ulcers and deep vein thrombosis (27). Finally, the exacerbation of cardiovascular comorbidities further strengthens this association. IS patients often have comorbidities such as hypertension, diabetes, and coronary heart disease. Chronic inflammation and malnutrition in the context of low PNI accelerate atherosclerosis progression, worsen cardiac function and metabolic disorders, and increase the risk of severe cardiovascular events (e.g., myocardial infarction, heart failure) post-stroke (28, 29). In summary, reduced PNI mediates the increase in all-cause mortality in IS patients by synergistically exacerbating neuroinflammation and immune dysregulation, oxidative stress injury, apoptosis and excitotoxicity, BBB disruption and secondary complications, while also worsening the prognosis of cardiovascular comorbidities. These mechanisms not only provide theoretical support for PNI’s prognostic value but also emphasize the importance of early nutritional intervention and immune regulation in IS management. Although these mechanisms partially explain the association, additional cellular experiments and animal model studies are needed to further validate specific regulatory targets.

While this study confirms the independent prognostic value of PNI for all-cause mortality in patients with IS, several limitations warrant acknowledgment when interpreting the findings. First, although this study included 1,152 consecutive patients, the study population was recruited exclusively from Northeast China, which may not reflect the real-world situation of other populations. The regional characteristics of the enrolled population should be considered when interpreting our results. Second, as a retrospective observational study, we only identified an independent association between PNI and all-cause mortality, rather than a definitive causal relationship, and unmeasured confounders not included in our adjusted models may have biased the observed associations. Third, the median follow-up duration of 14.23 months was relatively short, and we did not perform dynamic monitoring of PNI levels during follow-up, which limits the assessment of the long-term stability of PNI’s prognostic performance in IS patients. Finally, this study focused solely on all-cause mortality as the primary endpoint, without differentiating between stroke-specific mortality and death from other causes, nor systematically evaluating other IS-related adverse clinical outcomes. Meanwhile, the exclusion of patients with severe comorbidities may have introduced selection bias and restricted the generalizability of our findings to IS patients with multiple underlying diseases or organ dysfunction.

## 5 Conclusion

This study confirms that PNI is an independent prognostic biomarker for all-cause mortality in patients with IS, with higher PNI levels significantly associated with a reduced mortality risk. Clinically, PNI—derived from routine laboratory tests—can be used to stratify risk and guide the evaluation of nutritional and immune status in IS patients, supporting targeted interventions. Future research should integrate artificial intelligence, bioinformatics, computational medicine, and basic experiments to address the limitations of this single-center retrospective study, thereby facilitating the clinical translation of PNI-related strategies to reduce IS-associated mortality.

## Data Availability

The raw data supporting the conclusions of this article will be made available by the authors, without undue reservation.
